# Systems neuroscience: A box full of tools to illuminate the black box of the brain

**DOI:** 10.1371/journal.pbio.3002221

**Published:** 2023-07-27

**Authors:** J. Simon Wiegert, Marc Spehr, Ileana L. Hanganu-Opatz

**Affiliations:** 1 Department of Neurophysiology, MCTN, Medical Faculty Mannheim, Heidelberg University, Mannheim, Germany; 2 Department of Chemosensation, Institute of Biology II, RWTH Aachen University, Aachen, Germany; 3 Institute of Developmental Neurophysiology, Center for Molecular Neurobiology, Hamburg Center of Neuroscience, University Medical Center Hamburg-Eppendorf, Hamburg, Germany

## Abstract

Neurotechnological progress has greatly advanced our understanding of brain function over the past two decades. This Perspective looks at how novel technologies and tools have accelerated progress in systems neuroscience and discusses future strategies to elucidate brain function.

## Teaser

Investigation of brain function has been fueled by an accelerating development of novel technologies and tools. This Perspective looks at the unprecedented neurotechnological progress of the past 2 decades and discusses future strategies to elucidate brain function.

This article is part of the *PLOS Biology* 20th Anniversary Collection.

**Fig 1:**
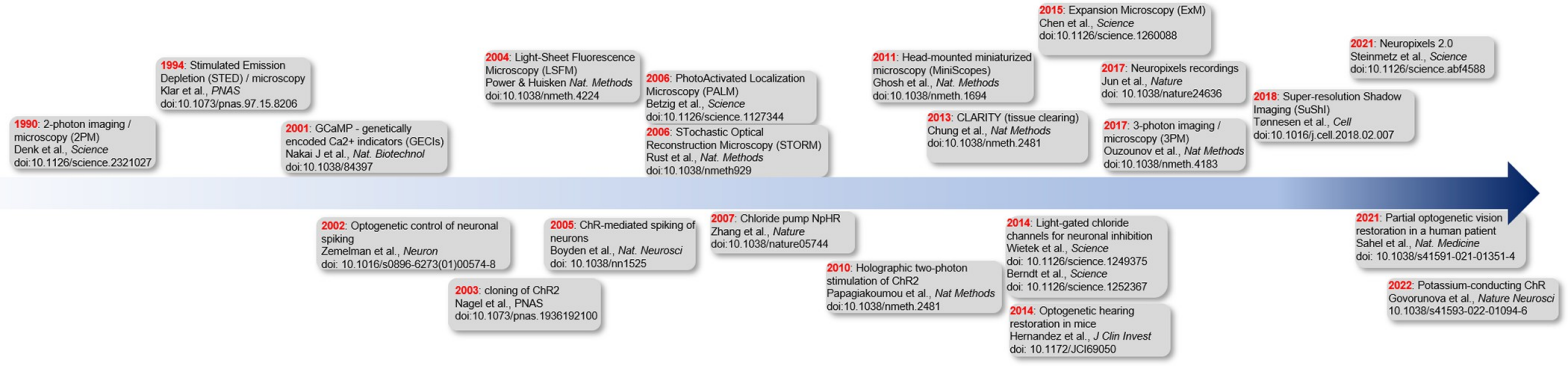
The development of tools to elucidate brain function. This timeline highlights the development of tools for examining brain function in a research setting. Although the focus of this Perspective is on developments from the past 20 years, some key tools from the preceding decade are also included.

Actions and thoughts are encoded in our brains. Cracking this code is a central aim of neuroscience but to do so requires access to the function of individual neurons as organisms perform behaviors. To monitor neuronal function, electrophysiological techniques with high temporal resolution [1] and imaging methods with high spatial resolution [2] have become standard methods that complement each other. However, technical limitations still hamper our ability to monitor brain activity in all its complexity. Consequently, huge collaborative efforts have been made during the past 2 decades to solve these problems, bringing together numerous national and international consortia of neurophysiologists, engineers, biophysicists, and theoreticians (e.g., Brain Initiative, International Brain Laboratory, and Allen Brain Map). These efforts have yielded unprecedented neurotechnological progress that, besides enriching fundamental research, have started (albeit rather sluggishly) to fuel clinical applications beyond cochlea implants and deep brain stimulation [3]. Here, we focus on tools developed in the past 20 years that have enhanced our ability to monitor brain function through recording brain activity, visualizing neuronal structures and manipulating specific cells (Fig 1).

In the past 20 years, electrophysiological monitoring of neuronal activity has been achieved extracellularly, juxtacellularly, and intracellularly, but an entirely new level of precision in understanding neuronal communication became possible following the development of Neuropixels probes in 2017 [4] and Neuropixels 2.0 in 2021 [5] (Fig 1). These probes were designed to enable better assessment of neuronal computations, both locally and at a large scale, through high-density recording site arrangement. Neuropixels probes allow functional dissection of behaviorally relevant multiarea brain networks at a cellular resolution. Impressive examples of their use include investigating the midbrain–thalamus–cortex circuit for movement initiation [6] and the cortex–basal ganglia–midbrain circuit for decision-making [7]. However, major computational challenges (e.g., data storage and reliable automatic spike sorting) and difficulties related to precisely localizing the probes will need to be considered and solved in the future. Moreover, the use of Neuropixels in primates and humans and, consequently, their clinical relevance is still rather limited [8,9].

The past 2 decades have also witnessed an increasingly dynamic evolution of transformative approaches to imaging the nervous system (Fig 1). The (re)appreciation that true insight into neural function depends reciprocally on an in-depth understanding of the underlying structure(s) has driven a surge in efforts to develop novel imaging techniques. In fact, keeping track of these approaches, which continue to emerge at a staggering pace, has become a challenge by itself. Visualization of the finest details of subcellular neuronal structure at an extremely high resolution has been achieved via various superresolution “nanoscopy” techniques [10]. While the necessary instrumentation comes with a substantial price tag, expansion microscopy provides a budget-friendly alternative. At the mesoscale, structural analysis has tremendously benefitted from the advent of tissue clearing techniques [11], which now allow the rendering of large tissue volumes optically transparent. Similar to Neuropixels recordings, the downside of these approaches is the immense volume of data generated by large-scale 3D imaging at a cellular resolution.

Imaging nervous system function benefits from the acquisition of spatial information and also from the ability to target specific neuronal (sub)populations. Over the past 20 years, genetically encoded Ca^2+^ indicators, particularly of the GCaMP brand, have become the gold standard for monitoring population activity [12]. Yet, all available Ca^2+^ reporters still suffer from relatively poor temporal resolution and limited linear dynamic ranges. In combination with head-mounted miniaturized microscopes, high-resolution Ca^2+^ imaging in deep and/or otherwise inaccessible brain areas of freely behaving animals becomes possible. Whichever tool neuroscientists choose to monitor neuronal activity, the ability to additionally exert precise control over neural discharge using optogenetics has enabled elegant manipulations that would have been deemed simply impossible 2 decades ago.

In 2003, the year *PLOS Biology* was founded, Nagel and colleagues published a paper [13] demonstrating that the algal protein channelrhodopsin-2 is a light-sensitive cation channel that can be expressed in mammalian cells. This discovery laid the grounds for modern optogenetics, which involves the expression of genetically encoded light-sensitive proteins in excitable cells such as neurons, so that their electrical activity can be manipulated with light. Together with efficient gene delivery methods based on genetically engineered mouse lines and viral vectors, precise optogenetic manipulation of defined neuronal populations has become a standard intervention in many disciplines in the neurosciences.

The ongoing discovery and engineering of new optogenetic tools with different spectral and kinetic properties, as well as ion or charge selectivity, has enabled a wide spectrum of manipulations, reaching from chronic activation and silencing to temporally precise multiplexed control of independent neuronal populations at a cellular resolution (Fig 1). Notably, optogenetics goes far beyond direct manipulation of neuronal activity, and the list of tools for light-mediated control of intracellular signaling, gene transcription, cytoskeletal dynamics, protein transport, and many more is constantly growing [14]. Thus, multispectral control of several modalities such as G-protein signaling and neuronal spiking will become more tangible in the future. Furthermore, the vast expansion of genetically encoded indicators for visualization of neuronal activity, second messengers, neurotransmitters, and more provides a bright perspective for all-optical investigations of neural communication and network function. However, most experimental configurations rely on synchronous wide-field stimulation of neurons, which provides an artificial condition, ignoring the importance of millisecond timing in neuronal communication. Combining fast and potent opsins with state-of-the-art 3D light shaping methods is desired and holds the potential for investigations of neural codes in living vertebrates in a more naturalistic setting. A future promise of optogenetics that has yet to be fulfilled is the disentanglement of instructive from permissive roles of particular brain areas for a particular behavior. Moreover, the use of optogenetics for medical interventions is an attractive future perspective, since it allows control of cellular activity in a spatiotemporally controlled manner, bypassing or overruling nonfunctional neuronal populations. Examples of possible future medical applications include vision and hearing restoration or closed-loop feedback control of aberrant brain activity.

Thus, the toolbox for examining brain function developed in the past 2 decades is yielding rich, multidimensional datasets of high complexity. The challenge for the future will be to transform this deluge of data into novel conceptual insights into brain function. To do so, several critical questions need to be addressed:

- How to “translate” the knowledge obtained with electrophysiological and imaging techniques? Imaging is limited by the kinetics of the genetically encoded indicators, while electrophysiology does not provide reliable spatial information about (sub)cellular locations. Bright voltage indicators with high dynamic range and high-density electrode arrays are minimizing the gap between the two technologies.
- How to deal with the drawbacks and limitations of (genetically encoded) Ca^2+^ indicators? Currently, we lack truly quantitative (ratiometric) reporters that come close to the signal-to-noise ratio achieved by intensity-based indicators, such as members of the jGCaMP8 family.
- Can we achieve multispectral control of neuronal populations with realistic spiking patterns? Most rhodopsins have broad action spectra, limiting their combined use to usually 2 opsins in the same experiments. Moreover, the kinetics of typical channelrhodopsins are one order of magnitude slower than natural ion channels, which leads to broadening of action potentials.
- Can optogenetic stimulation recapitulate naturalistic activity? Due to the synchronous activation of opsins and their kinetic properties, optogenetic stimulation is still highly artificial and does not represent the natural activity of neuronal networks. Thus, information may get strongly distorted, leading to wrong conclusions about the network under investigation.
- Can we develop tools to chronically monitor the brain activity in humans throughout life? Most of our knowledge on human brain activity is a result of EEG and/or combined EEG and MRI monitoring, which have the advantage of being noninvasive. However, they miss the fine temporal and spatial resolution of brain activity that is necessary for resolving the behaviorally relevant functional interactions between distinct neuronal types in different brain areas.
- Can we achieve medical interventions using optogenetics? Delivery and expression of optogenetic tools is still limited in humans. Long-term effects of overexpression are not yet well understood. In addition, gene delivery and light stimulation methods need to become more efficient—especially when targeting deep brain areas.

While it is a safe bet that methods will continue to improve, the next frontier in systems neuroscience will be to generate novel theoretical concepts of neural coding that can be proven (or falsified) by the experimental data we routinely accumulate using the fascinating new tools available today.
